# The Potential of Nanotechnology in Medically Assisted Reproduction

**DOI:** 10.3389/fphar.2017.00994

**Published:** 2018-01-11

**Authors:** Mariana H. Remião, Natalia V. Segatto, Adriana Pohlmann, Silvia S. Guterres, Fabiana K. Seixas, Tiago Collares

**Affiliations:** ^1^Biotechnology Graduate Program, Molecular and Cellular Oncology Research Group, Laboratory of Cancer Biotechnology, Technology Development Center, Federal University of Pelotas, Pelotas, Brazil; ^2^Post-graduation Program in Pharmaceutical Sciences, Federal University of Rio Grande do Sul, Porto Alegre, Brazil

**Keywords:** assisted reproductive technologies, nanotechnology, nanobiotechnology, multiple pregnancy, *in vitro* maturation, gene therapy, embryology

## Abstract

Reproductive medicine is a field of science which searches for new alternatives not only to help couples achieve pregnancy and preserve fertility, but also to diagnose and treat diseases which can impair the normal operation of the reproductive tract. Assisted reproductive technology (ART) is a set of methodologies applied to cases related to infertility. Despite being highly practiced worldwide, ART presents some challenges, which still require special attention. Nanotechnology, as a tool for reproductive medicine, has been considered to help overcome some of those impairments. Over recent years, nanotechnology approaches applied to reproductive medicine have provided strategies to improve diagnosis and increase specificity and sensitivity. For *in vitro* embryo production, studies in non-human models have been used to deliver molecules to gametes and embryos. The exploration of nanotechnology for ART would bring great advances. In this way, experiments in non-human models to test the development and safety of new protocols using nanomaterials are very important for informing potential future employment in humans. This paper presents recent developments in nanotechnology regarding impairments still faced by ART: ovary stimulation, multiple pregnancy, and genetic disorders. New perspectives for further use of nanotechnology in reproductive medicine studies are also discussed.

## Reproductive Medicine and Nanotechnology

Infertility and subfertility defined as the difficulty to conceive are conditions affecting people worldwide. The World Health Organization considers infertile couples those who fail to achieve a clinical pregnancy after, at least, 1 year of regular unprotected sexual intercourse (Zegers-Hochschild et al., 2017). Couples who experience these difficulties can turn to reproductive medicine technologies to help solve the problem. One of the most revolutionary treatments in this area is assisted reproductive technology (ART) comprising of *in vitro* embryo production (IVEP).

Regarding reproductive medicine, nanotechnology can be very useful in the development of non-invasive detection, diagnosis, and minimally invasive treatment of infertility-related disorders (oncological or non-oncological) (Barkalina et al., 2014a). To improve diagnostics, nanotechnology is applied mainly to the development and improvement of nanobiosensors and imaging techniques. Nanobiosensors are devices capable of identifying antigens, proteins, nucleic acids, and reactive oxygen and nitrogen species with quickness and sensitivity (Shi et al., 2007; Zhu et al., 2015). These technologies are underlying the development of interesting ‘lab-on-a-chip’ tools. Besides advantages of nanotechnology, this tool requires small volumes of analyte and reagents (Craighead, 2006; Hill and Li, 2017). The functionalization of zinc oxide nanoroads – gold nanoparticles (Gasparotto et al., 2017), iron oxide nanoparticles (Pal et al., 2015), and silica-coated gold nanoparticles with cadmium selenide quantum dots (Johari-Ahar et al., 2015) with anti-CA125 antibodies represent successful strategies to develop higher sensitivity tools for ovarian cancer detection. In addition, anti-HE4 antibody attached to silver nanoparticles was also used to develop a fast, specific, and stable ovarian cancer detection system (Yuan et al., 2012).

Biosensors using anti-PSA antibodies to detect PSA antigen represent one of the most used strategies for detection of prostate cancer. Gold nanoparticles functionalized with anti-PSA antibodies have been used in a bio-barcode assay showing ultrasensitivity (Thaxton et al., 2009) and in silicon nanowire field-effect transistors providing real-time prostate cancer detection (Presnova et al., 2017). Gold nanoparticles and anti-PSA antibodies supported in graphene oxide (Pal and Khan, 2017) or bound in cuprous oxide@ceric dioxide core–shell nanocomposites (Li et al., 2017) were also used to develop novel, accurate, and sensitive electrochemical immunosensors.

Diagnostic imaging has been improved by metallic and nanostructured particles, as these nanomaterials have great benefits compared to contrast agents. Iron oxide is one of the main contrast agents used for magnetic resonance imaging (MRI), and when nanostructured, it can be functionalized for additional benefits. Iron oxide nanoparticles can be formed in poly(vinyl alcohol), rendering them degradable over time (Bannerman et al., 2017) or, as showed in tumor xenograft animal models, can be associated to diatoms to improve tumor retention when a magnetic field is applied (Todd et al., 2014). In addition, iron oxide nanoparticles can be directed to a tumor site. For example, the functionalization of superparamagnetic iron oxide nanoparticles (SPIONs) with anti-prostate-specific membrane antigen (PSMA) increased the detection limit and the sensitivity of MRI in prostate tumor cell culture (Sillerud, 2016). In addition to iron oxide, other nanomaterials already tested in cell culture and/or animal models can be used as contrast agents to enhance imaging diagnostics, including gold nanoparticles (Indrasekara et al., 2013; Cole et al., 2015), carbon nanotubes (Liu et al., 2007; Vittorio et al., 2011), liposomes (Martina et al., 2005; Mukundan et al., 2006), dendrimers (Miyake et al., 2015), and quantum dots (Guo et al., 2014; Yao et al., 2016).

In the treatment of oncological diseases of the reproductive system, recent drug delivery and cell-target strategies have been developed. For example, one of the main anticancer drugs used, doxorubicin, has been associated to nanoformulations to increase its efficacy. These include mesoporous silica nanoparticles (Guo et al., 2017) and lipid-coated mesoporous iron oxide–based magnetic nanoassemblies (Pradhan et al., 2016) tested in human cell culture and xenograft mouse models, respectively. PEGylated liposomes have also been tested for cervical and ovarian cancer using human cells (Sriraman et al., 2016). Magnetic nanoparticles (Hua et al., 2017) have been used to treat cervical cancer in human cell cultures and xenograft mice. Other strategies include the delivering of siRNA in cationic dendritic starch (Engelberth et al., 2017), layer-by-layer engineering of upconversion nanoparticles (Lin et al., 2017), and mesoporous silica nanoparticles (Roberts et al., 2017) resulting in improved cell death in human ovarian cancer cells.

For non-oncological diseases of the reproductive system, some alternatives were tested in human cell culture. To treat uterine leiomyoma, strategies included the use of magnetic nanoparticles complexed to adenovirus (Shalaby et al., 2016) and nanoparticles loaded with 2-methoxyestradiol (Ali et al., 2013). In animal models, carbosilane dendrimer (Chonco et al., 2012) and nanoparticles-in-film (Cunha-Reis et al., 2016) were tested for the treatment of HIV infections. Another condition that could impair fertility is endometriosis, and the strategies already generated using nanomaterials are listed in **Table 1**.

**Table 1:** Strategies for treating endometriosis using materials in nanoscale.

Nanomaterial	Strategy	Animal model (cell type)	Main results	Bibliographic reference
Poly(lactic-co-glycolic acid) (PLGA) nanoparticles	PLGA nanoparticle to carry an anti-CD4 antibody	Female C57 endometriosis mouse model	The proposed treatment inhibited IL-10 and TGF-beta secreted by CD4+CD25+Treg cells.	Liu et al., 2017
Polyethylenimine-grafted chitosan oligosaccharide (CSO-PEI) with hyaluronic acid (HA)	Gene delivery of aquaporin 2 – small interfering RNA by polymeric nanoparticles	Ishikawa (IK) cells and female Sprague-Dawley rats with induced endometrial lesions	The tested strategy decreased the endometriotic lesion sizes with atrophy and degeneration of the ectopic endometrium. Also, the epithelial cells of ectopic endometrium showed a significant decrease of CD44 expression.	Zhao et al., 2016
Polyvinylpyrrolidone (PVP K-30)	Nanoencapsulation of copaiba oil-resin	Primary cell cultures of endometrial stromal cells (ESCs) obtained from ectopic endometrium of patients with endometriosis (EuESCs), ESCs obtained from ectopic endometrium of patients without endometriosis (CESCs) and ESCs from endometriotic lesions (EctESCs)	The proposed method reduced viability and proliferation of endometriotic cell cultures upon COPA nanocomposite treatment.	De Almeida Borges et al., 2016
Poly(lactic-co-glycolic) (PLGA) nanoparticles	Nanoencapsulation of epigallocatechin gallate and doxycycline	Human skin keratinocyte (HACAT) cell line and Swiss albino female mice	The proposed treatment decreased oxidative stress, matrix metalloproteinase activity, angiogenesis, endometrial gland presence and microvessel density, and improved oocyte quality.	Singh et al., 2015
Unmodified silica nanoparticles (UMNPs) and modified by aminopropyl groups silica nanoparticles (AMNPs)	Nanoencapsulation of glucosaminyl muramyl dipeptide (N-acetylglucosaminyl-N-acetylmuramyl-L-alanyl-D-isoglutamine) (GMDP)	Peritoneal mononuclear cells (MNC) derived from peritoneal fluid of women with endometriosis	The proposed strategy improved immunomodulatory effect of GMDP by the nanoencapsulation in silica nanoparticles.	Antsiferova et al., 2013
Cerium oxide nanoparticles (nanoceria)	Mitigation of endometrial lesions by nanoceria	CD-1 strain Swiss Albino female mice endometriosis induced	The nanoceria decreased oxidative stress, inhibited angiogenesis, and protected oocytes from endometriosis-related adverse effects.	Chaudhury et al., 2013
Chitosan-derived polymeric micelles with glycolipid-like structure	Gene delivery of pigment epithelium derived factor gene by micelles	Female Sprague-Dawley rats with induced endometrial lesions	The proposed gene therapy caused a decrease in the sizes of the endometriotic lesions, an atrophy and degeneration of ectopic endometrium, a significantly decrease in microvessel density and increased index of apoptotic in endometriotic lesions.	Zhao et al., 2012


In the field of fertility preservation, nanotechnology was shown to improve the potential of cryopreserved human immature testicular tissue to restore fertility. Dextran–chitosan nanoparticles loaded with vascular endothelial growth factor (VEGF) were tested for tissue engraftment after cryopreservation of the tissue in mice, resulting in higher vascular density and spermatogonia recovery in transplanted tissues (Poels et al., 2016). For female gametes, an interesting strategy for swine oocyte cryopreservation was developed. The addition of low concentrations of hydroxy apatite nanoparticles (less than 0.5%) in cryoprotectant agents increased the developmental rate of vitrified/devitrified germinal vesicles oocytes (Li et al., 2016). These are a few of the different contributions that nanotechnology has been giving to medically assisted reproduction.

## Potential Contributions of Nanotechnology to Assisted Reproductive Technology

Although ART is successfully applied as a clinical treatment worldwide, some challenges remain. Because of this, strategies developed in animal models are highly important for identifying new alternatives to overcome these problems. When it comes to ART, embryo development in mammalian models is highly similar to humans (Niemann and Wrenzycki, 2000; Barkalina et al., 2016). Lagomorph, murine, swine, bovine, and non-human primates are the main species used to study IVEP techniques to be applied to humans.

Similarly, the implementation of nanotechnology, which has already been developed for non-human animals, could be applied to assisted reproduction in humans (Langbeen et al., 2015; Barkalina et al., 2016). As mentioned previously, this technology has already been tested and used in sectors adjacent to reproductive medicine. Therefore, the main challenges of ART nowadays are how nanotechnology can intervene in order to boost the techniques already used today.

## Ovarian Stimulation and *In Vitro* Maturation

To perform ART procedures, ovarian stimulation is routinelly required in order to obtain a higher number of oocytes and increase the chances of embryo production to enable the selection of the best quality embryos for transfer (Fauser et al., 2005). Despite the increased number of oocytes that can be obtained using this procedure, some impairment has been observed. In addition to the high costs and the modest success rates, there are also potential health risks for the patients such as ovarian hyperstimulation syndrome in case of hyperresponse to ovarian stimulation (Huang et al., 2010).

*In vitro* maturation (IVM) is one of the most promising strategies for overcoming problems related to ovarian stimulation. Oocyte maturation consists of modification of genomic structures, organelle restructurations, and molecular production to allow the gamete to receive spermatozoa for fertilization (Fulka et al., 1998; Mao et al., 2014). Using the IVM technique, immature oocytes are collected from ovaries of non-stimulated patients, followed by selection and exposure to IVM medium consisting of a base medium for cell culture supplemented with hormones, including FSH, LH, and estradiol. However, despite its clinical utility and successfull application in farm animals (Goto et al., 1988; Hwu et al., 1998), IVM of human oocytes remains an experimental approach not widely accepted in fertility clinics worldwide (Chang et al., 2014; Tannus et al., 2017). This is likely due to the lower pregnancy and live birth rates using *in vitro* compared to *in vivo* maturated oocytes, likely due to inadequacies of the culture media (Combelles et al., 2002; Ortega-Hrepich et al., 2013).

It is well established that embryo quality is dependent on oocyte quality (Lonergan et al., 2003; Ferris et al., 2016). In addition, correct and complete oocyte maturation is essential to efficient embryo production. Regarding IVM, the process can be disrupted by excess production of ROS, which is one of the major causes of oocyte depletion (Tamura et al., 2008; Karuputhula et al., 2013). For IVM, the addition of antioxidants is helpful, but these molecules may not exert their function with high efficiency due to their instability in *in vitro* enviroment, making utilization of nanomaterials an interesting strategy for molecule protection (Lucas et al., 2015; Komninou et al., 2016; Remião et al., 2016; Duarah et al., 2017; Manconi et al., 2017). One study from our group has show increased cleavage and blastocyst production rates, decreased ROS levels, and decreased the number of apoptotic cells/blastocyst when bovine oocytes were supplemented with nanoencapsulated melatonin in a IVM medium (Remião et al., 2016).

In another study, tretinoin was nanoencapsulated in lipid-core nanocapsules (LNC) and supplementation with the minor tested concentration (0.25 μM) in IVM medium was benneficial for bovine oocytes, resulting in higher cleavage and blastocyst rates, decreased P66Shc protein levels (the 66-kDa isoform of the growth-factor adapter Shc), and decreased ROS production. These benefits were not observed using the same concentration of non-encapsulated tretinoin (Lucas et al., 2015). Therefore, this represents a potential strategy for increasing the effectivness of human IVM and IVEP.

## Multiple Pregnancy

Multiple pregnancies are a current problem in ART. The incidence of multiple pregnancies is related to pre-term birth, birth of babies with low weight and other complications, and risks to mothers and babies (Fauser et al., 2005; Vulliemoz et al., 2012). The high incidence of multiple pregnancies when using ART is related to the fact that sometimes more than one embryo is transferred into the female reproductive tract (Friedman et al., 2011; Mersereau et al., 2017).

In order to overcome the multiple pregnancy problems in ART, one alternative is the transfer of single embryos performed at a higher frequency (Mancuso et al., 2016). The methodologies assisting this condition are IVM, *in vitro* blastocyst culture, and embryo cryopreservation, techniques that have been highly studied in small and large animals and have been utilized commercially for many years (Sinclair, 2008).

Preimplantational genetic screening (PGS) and preimplantational genetic diagnosis (PGD) can also be useful to avoid multiple pregnancies, by discarding embryos with genetic disorders. To perform PGS and PGD, embryos are biopsied and evaluated using techniques such as karyotyping, fluorescent *in situ* hybridization (FISH), quantitative polymerase chain reaction (qPCR), array comparative genomic hybridization (aCGH), and next generation sequencing (NGS) (Chen et al., 2017).

Nanotechnology can help researches improve the application of PGS and PGD. Although highly employed, the current detection methods could be more sensitive and specific, more affordable and accessible to patients, faster, and easier to use to facilitate use in human reproduction clinics. Gold, silver, carbon, and magnetic nanomaterials are the main materials used to develop new methods of genetic diagnostics (Zhu et al., 2015). Nanotechnology combined with colorimetric (Stoeva et al., 2006) and electrochemical (Ozsoz et al., 2003) methods for nucleic acid analysis and detection has brought more sensitivity, lower cost, and increased simplicity and portability to diagnostics. This and other strategies recently developed for DNA analysis can be applied in the future to simplify PGD and PGS diagnostic procedures.

Another strategy for embryo selection is the culture of human embryos until day 5/6, when they reach the blastocyst stage. It has been previously shown that blastocyst transfer (day 5/6) presents better results than cleavage embryos (day 2/3) (Abuzeid et al., 2014; Yin et al., 2017). However, some clinics transfer embryos at the cleavage stage because most embryos fail to reach day 5/6 due to difficulties in mimicking the complexities of the *in vivo* environment (Alper et al., 2001; Tsirigotis, 1998). *In vitro* culture and manipulation of gametes and embryos stimulates production of exogenous ROS and leads to oxidative stress, reducing embryo quality (Agarwal et al., 2006; Truong et al., 2016). To overcome the challenge of embryo culture leading up to the blastocyst stage, research groups have looked for alternative approaches to improve *in vitro* embryo culture, including the addition of antioxidant molecules to the medium.

Studies on IVEP in animal models indicate antioxidant supplementation in medium is beneficial for blastocyst production. Antioxidants presenting beneficial effects in animal model *in vitro* embryo cultures include L-carnitine (Abdelrazik et al., 2009), hyaluronan (Romek et al., 2017), resveratrol (Salzano et al., 2014), and melatonin (Wang et al., 2013, 2014). However, in the case of bovine IVM, nanotechnology provides interesting alternatives for protecting of these molecules in *in vitro* environments (Lucas et al., 2015; Komninou et al., 2016; Remião et al., 2016). A recent publication confirmed this approach may represent a relevant alternative: supplementation of IVC medium with melatonin-loaded LNC increased embryo quality and blastocyst hatching in a bovine model (Komninou et al., 2016). This strategy is beneficial since the nanocapsules are biodegradable and do not result in toxicity when exposed to bovine oocytes (Lucas et al., 2017) or administered intradermally in rats (Bulcão et al., 2014).

## Genetic Disorders

The development and improvement of genome editing technology in the last few years has introduced gene therapy as a pre-emptive solution for correction of genetic anomalies. Monogenic diseases may be easily corrected using gene therapy, as they are caused by a single defective gene (Ma et al., 2017). Some monogenic diseases have already been targeted by gene therapy techniques, including lipoprotein lipase deficiency (Gaudet et al., 2016), hemophilia B (Nathwani et al., 2017), β-hemoglobinopathies (Negre et al., 2016), Wiskott–Aldrich syndrome (Aiuti et al., 2013; Morris et al., 2017), and inherited retinal degenerations (Gupta and Huckefeldt, 2017), although these diseases have not been treated in embryos.

Two recent reports have already shown the possibility of gene editing human embryos to correct genetic disorders. The studies used the CRISPR-Cas9 method to fix the human β-globin gene (Liang et al., 2015) and heterozygous *MYBPC3* mutation (Ma et al., 2017), mutations responsible for β-thalassemia and hypertrophic cardiomyopathy, respectively. Although these studies have raised ethical concerns and the technology is still experimental, without proven efficacy and safety, both publications bring an important alternative to reproductive medicine through the treatment of diseases that until now were considered incurable (Ishii, 2017).

Nanotechnology development has resulted in some interesting non-viral strategies for molecule delivery in cells (Barkalina et al., 2015) contributing to the optimization of gene editing. One of them is the study of Sun et al. (2015) that delivered the Cas9 protein and a guide RNA through a DNA nanoclew to human osteosarcoma tumors in mice. Diverse studies have shown efficient gene delivery in mammalian cells via nanomaterials (Guan and Rosenecker, 2017; Riley and Vermerris, 2017). To produce genetically modified embryos, nanomaterials can be used to increase the efficiency of gene transfer via sperm mediated gene transfer. Silica nanoparticles (Barkalina et al., 2014b), magnetic iron nanoparticles (Kim et al., 2010), halloysite clay nanotubes (Campos et al., 2011), and poly(vinyl alcohol)-coated iron oxide nanoparticles (Makhluf et al., 2008) have already shown promise for delivery of nucleic acids and/or proteins to bovine spermatozoa.

Single-cell embryos can also be directly modified using nanomaterials. Das et al. (2016) hypothesized that if single-cell stage zona-free bubaline embryos are transfected with commercial transfecting agents and developed until the blastocyst stage (Selokar et al., 2015), nanomaterials could also be used to introduce genes into embryos at this stage and condition. This could be an alternative to not only viral vectors, but also other expensive methods such as pronuclear microinjection (Das et al., 2016). However, more studies are needed before introducing this technology into practice due to the possible toxic effects.

## Challenges for the Use of Nanotechnology in Reproductive Science

Nanotechnology has already and can continue to provide advantages for reproductive medicine. Despite the great solutions it can offer, some challenges still faces the use of this technology in medicine. As it is an emerging science, few studies have been performed to validate all the possibilities for treatments or diagnostics. One of the main questions that still need to be addressed regarding the use of nanotechnology is the toxicity it could cause. Despite new diagnostic methodologies being closer to being applied commercially, the ways in which nanomaterials are administered to organisms, embryos, or gametes need to be further studied.

Some nanomaterials are toxic to organism, mainly when exposure occurs during pregnancy and embryo development. For example, when pregnant mice are exposed to titanium dioxide nanomaterials, these materials can cross the placental barrier and cause anatomical defects in the fetuses (Melnik et al., 2013; Naserzadeh et al., 2017). In addition, silver nanoparticles decrease oestrogen plasma levels, increasing the number of resorbed fetuses (Campagnolo et al., 2017) and affecting embryonic growth (Austin et al., 2016). Carbon nanotubes also decrease the number of live fetuses per dam (Fujitani et al., 2015), the number of blood vessels on placenta, and increase the number of abortions (Qi et al., 2014).

Because of this, the utilization of these nanomaterials for reproductive proposes should be done carefully. One alternative is to search for additional nanomaterials that do not present toxicity, with biodegradable structures as the LNC and the dextran/chitosan nanoparticles, representing the most promising nanostructures for use in health applications.

## Final Considerations

Despite the recent advances in assisted reproductive technologies, some challenges remain, mainly related to pregnancy rates, multiple births, and genetic disorders. To overcome these problems, new alternatives must be identified. Nanotechnology represents a valuable tool that must be explored further to help researchers identify solutions for reproductive medicine. Nanomaterials can bring specificity, practice, and sensibility to next-generation diagnostic and treatment modalities.

It is expected that, as in other areas of medicine, the employment of nanotechnology could be helpful and beneficial to patients. In addition, researchers must be encouraged to develop more *in vitro* and *in vivo* tests using animal models to test safety and efficiency of these new methodologies. In addition, human clinical reproductive trials may also help accelerate commercial availability of new alternatives for ART.

## Author Contributions

MR, NS, AP, SG, FS, and TC had an equal participation in writing and approving the present manuscript.

## Conflict of Interest Statement

The authors declare that the research was conducted in the absence of any commercial or financial relationships that could be construed as a potential conflict of interest.
